# Characteristics of an Extrusion Panel Made by Applying a Modified Curing Method

**DOI:** 10.3390/ma9050347

**Published:** 2016-05-07

**Authors:** Haseog Kim, Sangki Park, Seahyun Lee

**Affiliations:** Korea Institute of Civil Engineering and Building Technology, 283, Goyang-daero, Ilsanseo-gu, Goyang-si, Gyeonngi-do 10223, Korea; bravo3po@kict.re.kr (H.K.); shlee@kict.re.kr (S.L.)

**Keywords:** carbon dioxide, steam curing, ground granulated blast furnace slag, ladle furnace slag

## Abstract

CO_2_ emitted from building materials and the construction materials industry has reached about 67 million tons. Controls on the use of consumed fossil fuels and the reduction of emission gases are essential for the reduction of CO_2_ in the construction area as one reduces the second and third curing to emit CO_2_ in the construction materials industry. In this study, a new curing method was addressed by using a low energy curing admixture (LA) in order to exclude autoclave curing. The new curing method was applied to make panels. Then, its physical properties, depending on the mixed amount of fiber, type of fiber and mixed ratio of fiber, were observed. The type of fiber did not appear to be a main factor that affected strength, while the LA mixing ratio and mixing amount of fiber appeared to be major factors affecting the strength. Applying the proposed new curing method can reduce carbon and restrain the use of fossil fuels through a reduction of the second and third curing processes, which emit CO_2_ in the construction materials industry. Therefore, it will be helpful to reduce global warming.

## 1. Introduction

CO_2_, which accounts for the largest amount of greenhouse gases emitted through human activities, increased from about 290 ppm in the 19th century to 315 ppm in 1958 and finally reached 384 ppm in 2007. An annual growth rate of 2 ppm on average has been shown since 2000 [1]. All over the world, CO_2_ emissions from cement production account for 3% of total carbon emissions. Typically, approximately 900 kg of CO_2_ have been generated during the production of one ton of cement. In 2012, China’s average carbon dioxide emissions of 7.1 tons CO_2_ per capita was mainly driven by the increase in building construction and expansion of infrastructure, as indicated by the growth in cement and steel production [2]. Thus, the reduction of using cement will be helpful to reduce the CO_2_ emissions, which is one of the main reason for global warming [3].

In the construction field, which accounts for 42.3% as a source of CO_2_ emissions in Korea, CO_2_ emitted from building materials and the construction material industry has reached approximately 67 million tons, which totals about 30% of all CO_2_ arising from the construction field. In general, CO_2_ generated from the construction material industry mostly results from the combustion of fossil fuels used for the second and third curing performed after curing at room temperature for the purpose of acquiring target performance, developing early strength, increasing productivity and obtaining easy workability after the forming of products. It should be noted that curing is the process in which the concrete is protected from loss of moisture and kept within a reasonable temperature range. The result of this process is increased strength and decreased permeability [4].

As one of the typical dry walls, the cement extrusion panel has many advantages, such as being increasingly lightweight compared to its strength, dimensional stability, constructability, a strong resistance to moisture, and more. However, it has disadvantages. For instance, it requires greater cement consumption compared to silica sand, uses expensive raw materials, such as fibers and thickening agents, and needs the secondary steam curing and tertiary autoclave curing after forming. Due to these defects, its production cost is relatively high compared to competing products, including gypsum board and lightweight concrete panels. Therefore, its use is restricted.

Extrusion molding is a method of molding by passing input materials through a die when in a plastic state, and it is one of the most common production techniques for the production of plate and rod materials, as well as other linear members. Due to the extrusion molding method characterized by a continuous production system, it is often thought of as the most advantageous method in terms of productivity and economic efficiency and, moreover, has advantages in that it is relatively lightweight compared to wet pre-cast products and has higher strength in comparison to its mass, dimensional stability and moisture resistance [5]. However, due to its low commercial viability, caused by the use of expensive admixtures and an increase in production costs resulting from the second and third curing, it still has a small market share, and therefore, research is currently in progress to improve its economic efficiency and enhance the overall functionality.

Kim [6] improved the economic efficiency of the extruded panels using stone dust sludge and waste concrete power, while Choi [7] developed panels with improved fire resistance performance by mixing alpha-hemihydrate gypsum into the extrusion panel. These studies were aimed at reducing production costs in the manufacturing phase by mixing ordinary Portland cement and recycled materials as binding materials. It should be noted that the recycled material is a sort of slag, and the binding material is a sort of calcium sulfate aluminate binding material.

In order to solve this problem, this study sought to address this by using rapid hardening hydraulic cement instead of ordinary Portland cement as a binder for the exclusion of autoclave curing. The rapid hardening binder, which is a sort of inorganic chemical additive, has the characteristic that its hardening rate is relatively fast after losing fluidity over time and after concrete placement. In other words, the surface area of cement particles is enlarged by increasing the fineness of the cement to enhance the activation of the cement, and thus, the hydration reaction becomes more active when applied with water. This not only increases its speed, but the speed is also increased by changing the ingredients added to the binder [8,9,10].

In this regard, a comparative analysis on the strength and characteristics with a specimen subjected to the tertiary autoclave curing was conducted. A specimen consisted of the ground granulated blast furnace slag, calcium sulfate aluminate, ladle furnace slag, calcined kaolin, glauberite and fluosilicate salt. During the analysis, the added amount of calcined kaolin, glauberite and fluosilicate salt is fixed. The cement binder is substituted with the ground granulated blast furnace slag, calcium sulfate aluminate (CSA) and ladle furnace slag. Additionally, the optimum composition ratio was derived from the analysis results. Typically, slag is produced during the steel manufacturing process and is commonly used in concrete due to its effect of improving durability and the interface with the aggregate. Tam *et al.* [11] have conducted an experimental test with 0%, 20% and 100% of recycled aggregate substitutions to compare the durability. Their test have demonstrated that a two-stage mixing approach can help to improve the durability of recycled aggregate concrete. Thomas *et al.* [12] have evaluated the durability and mechanical properties of recycled concrete. A total of 24 mixtures at ages of 28, 180 and 365 days was considered. The durability of the concretes made with recycled aggregate is worse due to their intrinsic porosity. Moreover, the energy savings and resource-conserving properties can be obtained using slag-blended cement [13,14]. Gesoğlu and Özbay [15] have studied the use of granulated blast furnace slag, and the fresh and strength properties of concrete can be obtained by using the slag as a cement replacement material. Thus, the possibility of excluding the autoclave curing was confirmed by applying the developed binder to the cement extrusion panel [16,17,18].

Kim *et al.* [16] have conducted a test for manufactured mortar by having cement as the plain and substituting three binding materials. Different curing methods were considered to analyze congelation and strength characteristics. Test results for the strength property by changing binding materials showed that specimens with blast furnace slag, CSA 15% and CAMC (calcium aluminate modified composites) 5% resulted in a positive effect on strength improvement. Kim and Lee [18] have made an extrusion panel by using cement as the base material and substituting binding materials to analyze the strength characteristics. Their results showed that the strength of the extrusion panel improved when the replacement binder (low energy curing admixture) is used.

Accordingly, the main goal of this study is to address a new curing method by using a low energy curing admixture in order to exclude autoclave curing. The experimental test has been performed to evaluate the physical properties of the extrusion panels, which are manufactured by using the proposed curing method. Furthermore, this study aims to reduce carbon and restrain the use of fossil fuels through a reduction of the second and third curing processes, which emit CO_2_ in the construction materials industry, as part of the efforts to reduce CO_2_ in the construction field, which accounts for a high proportion of CO_2_ emissions in Korea, and so, to keep pace with domestic greenhouse gas reduction policies. Towards this end, it investigated the optimum mix of the extrusion panel using a low energy curing admixture (LA).

## 2. Background for Experiments

Orthogonal array designs [19], proposed by Dr. Genichi Taguchi, used to investigate the binder mixing ratio, fiber type and fiber mixing ratio are methods of experimental planning in which the number of test arrays varies depending on the presence of interactions. For instance, if there is no interaction, effective factors are determined by reducing the number of experiments at the expense of information concerning the factors affecting the results; and if there is an interaction, an experiment on the entire test group is conducted to determine effective factors. In this study, a three-level system, mainly used in cases where factors are measured values, was used as represented in Equation (1) [20].

(1) $$L_{3m}=3^{\left(\frac{3m-1}{2}\right)}$$

where *L* is a phase indicating the orthogonal array; *m* is an integer number of experiment factor; 3*m* is the size of the experiment; and (3*m* − 1)/2 is the number of columns of the orthogonal array.

### 2.1. Experimental Plans and Methods

In this study, an experiment was planned (summarized in Table 1) to derive the optimum mix by applying LA, which is the outcome of the previous research [16,18], for the extrusion panel. It should be commented that the ratio in Table 1 is the mass ratio. Additionally, the replacement ratio of LA is the ratio by dry components, and the replacement ratio of fiber is the ratio by the whole mixture. For the extrusion panel, fibers are used for the purpose of increasing the flexural strength and crack suppression, but only such fibers as PP (polypropylene) with a high heat resistance that ensure non-combustion during the high-pressure autoclave curing process should be selectively used; this, in turn, leads to the problem of rising production costs with respect to the expensive materials. The characteristics of fibers used in this study are summarized in Table 2.

Accordingly, since the autoclave curing process was excluded with the use of LA in this experiment, the applicability of PVA (polyvinyl acetate) and nylon fibers, which boast relatively low costs, was investigated to increase the flexural strength and to suppress cracks. Table 1 shows a summary of the experimental plan, where the LA replacement rate, fiber type and fiber mixing ratio were set as experimental factors, and Table 3 shows the experimental mixture.

**Figure 1:**
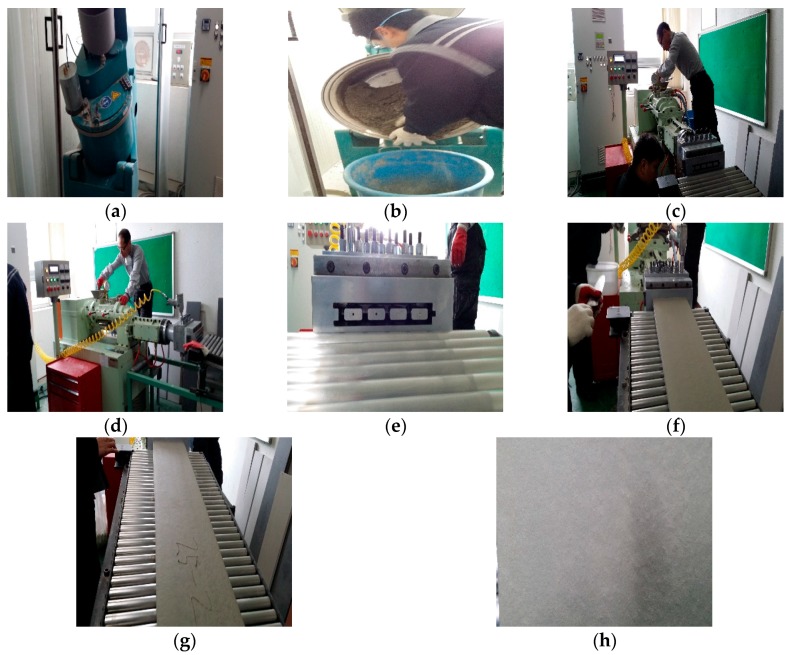
Production method of extrusion panel: (**a**) mixer; (**b**) mixing materials; (**c**) setting of extrusion machine; (**d**) input materials; (**e**) extrusion; (**f**) correction of horizontal; (**g**) manufacturing of panel; (**h**) surface of extrusion panel.

The extrusion specimen was produced in the form of a panel with sizes measuring 300 mm × 1000 mm × 35 mm by using an extruder of the SY-05S model by Ishikawa-toki Co., Tokyo, Japan, and the experimental method is shown in Figure 1. The flexural and compressive strengths of the extruded specimen, which was cut to 120 mm in length, were measured after the atmospheric pressure steam curing using a universal testing machine according to KS L ISO 679 [21], which is very similar to ISO 679 [22], and the absorption rate, moisture content and density of the panel were also measured according to KS F 4735 [23]. It should be commented that the specimens was molded at −1 atmosphere, and measurements have performed at 7 days after the curing process to evaluate the moisture content, the absorption ratio, the density, the flexural strength and the compressive strength. The water-binder (W/B) ratio for extrusion specimens is 12%. A binder having rheology is needed to manufacture the extrusion specimens. In this study, 50,000 CP (centipoise) viscosity agent is considered to improve the rheological property of the binder. However, the flow of the binder is close to almost zero. Therefore, it is impossible to measure the viscosity of the binder.

### 2.2. Curing Methods

**Figure 2:**
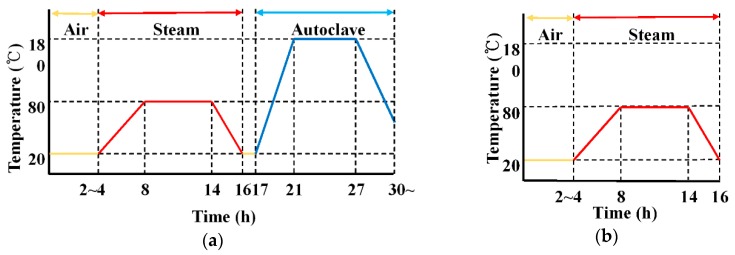
Curing process in this study: (**a**) traditional two-step curing process; (**b**) modified one-step curing process.

For the curing of the specimen molded by extrusion, room temperature curing and atmospheric pressure steam curing were performed, as shown in Figure 2, and high-pressure steam curing was performed for the base in which only cement was used. The curing at room temperature was performed in a constant temperature and humidity chamber under temperatures of 20 ± 1 °C, RH (relative humidity) 60% ± 5% conditions. For the atmospheric pressure steam curing, a method of natural cooling after rising for four hours and maintaining this for five hours under conditions of 80 °C, RH 100%, was used. For the high-pressure steam curing, a method of natural cooling after rising for four hours and maintaining this for five hours under conditions of 180 °C, 10 pressure, RH 100% was used with respect to the specimen steam cured at atmospheric pressure.

### 2.3. Used Materials

For cement, the ordinary Portland cement (Grade 1) available from Ssangyoung Cement Co., Seoul, Korea was used, and the ground granulated blast furnace slag used is Grade 3 ground granulated blast furnace slag generated as a by-product during the pig iron process, of which the specific surface area (Blaine) is 7400 cm^2^/g for the product from Hankook Slag & Materials Co., Seoul, Korea. As for the CSA (calcium sulfated aluminate), the specific surface area (Blaine) of a product of Hankook Slag & Materials Co., Seoul, Korea is 4500 cm^2^/g, and the product which contains 36.5% of Al_2_O_3_ was used.

Calcined kaolin is made of kaolinite calcined during the metakaolin manufacturing process. Although it exhibits lower reactivity compared to metakaolin, the product, which has a specific surface area (Blaine) of 4200 cm^2^/g, manufactured by Nycontech Co., Asan, Korea, was used.

CAC (calcium aluminate compound) is made by crushing the atomized ladle furnace slag into smaller particles with the specific surface area (Blaine) of 5000 cm^2^/g, and it is of similar composition to the CA-based ultra-rapid hardening chemical composition and characterized by a high hydration heat and rapid setting. In addition, glauberite and fluosilicate salt were used to contribute to the pozzolanic reaction of the slag and calcined kaolin.

These two materials are known to be helpful in improving durability, including crack suppression, suppression of the rise in hydration heat, neutralization and freeze-thaw resistance by being transferred to the soluble silica by hydrolysis in the cement matrix and promoting the pozzolanic reaction to react with the calcium hydroxide generated by cement hydration reactions [24]. The characteristics of each material are shown in Table 4, Table 5 and Table 6.

In addition, as a thickening agent used for controlling viscosity and improving the lubricating properties in the extrusion process, a product made by Samsung Fine Chemicals Co., Seoul, Korea with a viscosity of 40,000 cps and a density of 1.26 g/cm^3^, was used. Inorganic-based wollastonite and organic-based pulp were used as fiber materials in order to improve the tensile strength during the extrusion, and synthetic fibers, such as PP (polypropylene), PVA and nylon were also used.

## 3. Experimental Test Results

In total, 27 specimens were constructed by using an extruder for each combination. Specimens were cured for three days, and experimental tests were carried out to evaluate the characteristics of each extrusion panels. Results are summarized in Table 7. Detailed information is followed.

### 3.1. Moisture Content Ratio

**Figure 3:**
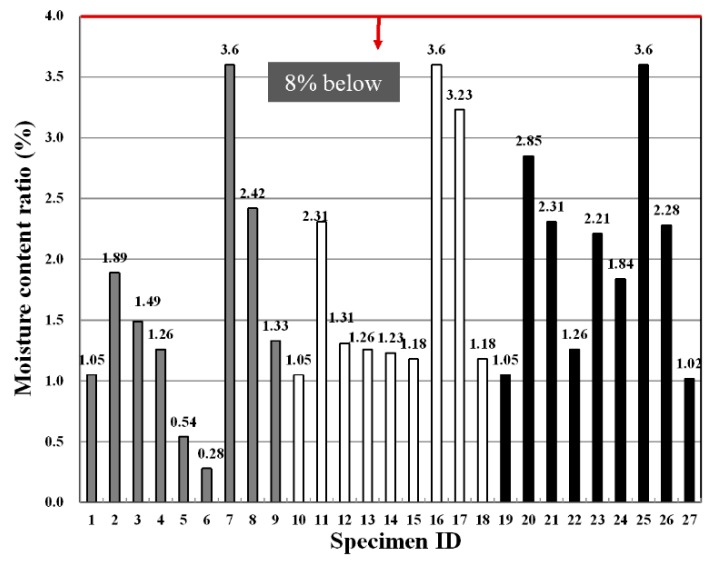
Variation of the moisture content ratio for specimens.

Figure 3 shows the moisture content ratios according to each fiber type, the fiber mixing ratio and the binder replacement rate, and they were found to comply with the standards in all experimental groups. The correlation of the moisture content according to fiber type, fiber mixing ratio and binder replacement rate was not found, and the moisture content showed a relatively high value in the specimen without fibers.

This suggests that, as reported in the existing literature, fibers are distributed inside the panel matrix to fill the fine pores and thus reduce the pathway of moisture and, thereby, degrading the moisture content [25]. In general, it is known that a reduction in the strength of the extrusion panel is due to cracks and warping caused by the drying shrinkage in a high volume of moisture content. Therefore, a low water content is considered to be advantageous in terms of physical performance and durability. IBM SPSS Statistics (V19.0, IBM Corp., Armonk, NY, USA, 2015) [26], the commercial statistics software package, is selected to perform the F-test in this study. Table 8 shows F-test results for each experimental factor obtained from an analysis of variance of moisture content measurement values. The results of the analysis of variance revealed that F-values of experimental factors like A (binder replacement rate), B (fiber type) and C (fiber mixing ratio) were 4.158, 25.490 and 11.208, respectively. Additionally, since the experimental values were two in degrees of freedom and 12 in error, thus critical values F_0.01_, F_0.05_ and F_0.10_ were 6.93, 3.89 and 2.81 at confidence limits of 99% (significance level α = 0.01), 95% (significance level α = 0.05) and 90% (significance level α = 0.10), respectively. Therefore, B (fiber type) and C (fiber mixing ratio) from among the three experimental factors were found to satisfy the 99% (significance level α = 0.01) level and turned out to be the most influential factors. In addition, A (binder replacement rate) achieved a 95% (significance level α = 0.05) level and, thus, was found to affect the base panel moisture content. A look at the changes in moisture content due to the interaction revealed that as the degree of freedom is four and the error is 12 and, thus, critical values F_0.01_, F_0.05_ and F_0.10_ were 5.41, 3.26 and 2.48 at confidence limits of 99% (significance level α = 0.01), 95% (significance level α = 0.05) and 90% (significance level α = 0.10), respectively, the interactions of A (binder replacement rate) and B (fiber type) among the three experimental factors were found to not be significant even at 90% (significance level α = 0.10). The influence of the interaction between A (binder replacement rate) and C (fiber mixing ratio) on the moisture content totaled was at the 99% (significance level α = 0.01) level, and there was no interaction between B (fiber type) and C (fiber mixing ratio).

**Figure 4:**
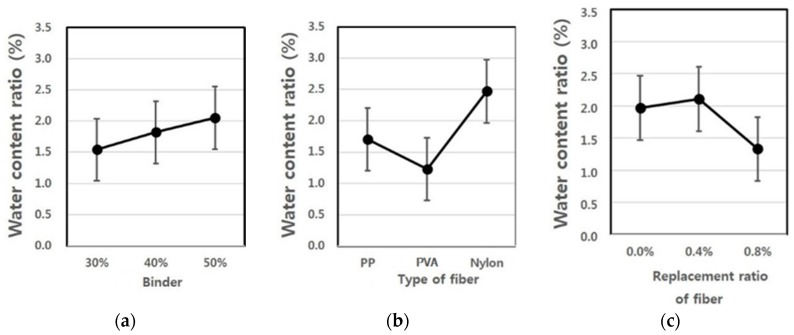
Assumed moisture content ratio of extrusion panel by variance analysis: (**a**) replacement ratio of binder; (**b**) type of fiber; (**c**) replacement ratio of fiber.

Figure 4 shows the moisture content values estimated using the test results, and the average value of the moisture content ranged from 1.2 to 2.5. As shown in Figure 4, an increase in the binder mixing ratio increased the moisture content, and an increase in the fiber mixing ratio resulted in the opposite. The use of nylon as a fiber showed the highest moisture content, and the specimen using PVA fiber exhibited the lowest moisture content value.

### 3.2. Dry Density

**Figure 5:**
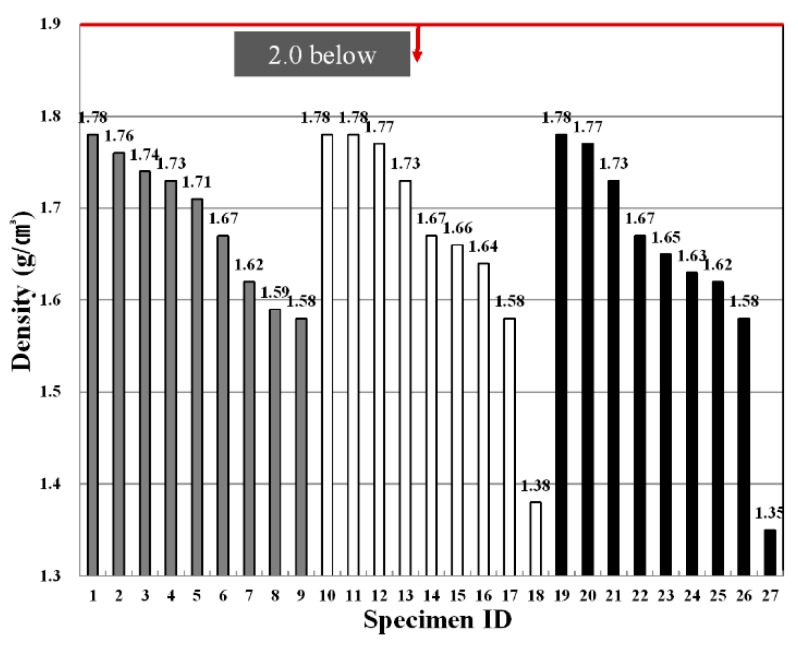
Variation of density for each specimen.

The densities depending on the fiber type, fiber mixing ratio and binder replacement rate were found to comply with standards in all experimental groups, as shown in Figure 5. A change in the densities was not affected by the fiber type, whereas the density changed according to the binder replacement rate and fiber mixing ratio. In particular, as the fiber mixing ratio and binder replacement rate increased, the density decreased. This is because the fiber used in the experiment consisted of a fairly light material with a specific gravity ranging from 0.2 to 0.4. The binder also had lower specific gravity values of 2.5–2.6 compared to the specific gravity of cement (3.06), and therefore, as the mixing ratio increases, the density of matrix decreases. Accordingly, specimens 3, 6, 9, 12, 15, 18, 21, 24 and 27 with higher fiber mixing ratios and greater binder replacement rates were found to exhibit the lowest density values. Higher densities pose difficulties in construction due to an increase in unit loads, whereas specimens with a relatively low density are considered to be advantageous in terms of construction.

Table 9 shows F-test results for each experimental factor obtained from an analysis of variance of density values. The results of the analysis of variance revealed that F values of experimental factors like A (binder replacement rate), B (fiber type), C (fiber mixing ratio) were 2.462, 59.331 and 11.375, respectively.

Additionally, since the experimental values were two in degrees of freedom and 16 in error and, thus, critical values F_0.01_, F_0.05_ and F_0.10_ were 6.23, 3.63 and 2.67 at confidence limits of 99% (significance level α = 0.01), 95% (significance level α = 0.05) and 90% (significance level α = 0.10), respectively, the experimental factors B (fiber type) and C (fiber mixing ratio) were found to satisfy the 99% (significance level α = 0.01) level and turned out to be the most influential factors. Meanwhile, A (binder replacement rate) was not significant even at 90% (significance level α = 0.05) and, thus, was found to have little influence on the changes in density values.

**Figure 6:**
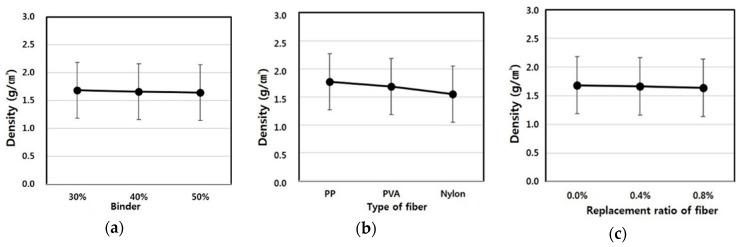
Assumed dry density of the extrusion panel by variance analysis: (**a**) replacement ratio of binder; (**b**) type of fiber; (**c**) replacement ratio of fiber.

A look at the changes in densities due to the interaction revealed that as the degree of freedom is four, and the error is 16 and, thus, critical values F_0.01_, F_0.05_ and F_0.10_ were 4.77, 3.01 and 2.33 at confidence limits of 99% (significance level α = 0.01), 95% (significance level α = 0.05) and 90% (significance level α = 0.10), respectively, there was no interaction of A (binder replacement rate), B (fiber type) and C (fiber mixing ratio). However, there was a change in density due to an interaction between B (fiber type) and C (fiber mixing ratio), and its effects was found to meet 95% (significance level α = 0.05). Figure 6 shows the density values estimated using the test results, and the average value of the density ranged from 1.5 to 2.0. As shown in the figure, an increase in the binder mixing ratio had no influence on the change in density, whereas the density values were found to change depending on the increase in the fiber mixing ratio and fiber type. The use of nylon as a fiber showed the lowest density value, and the specimen using PVA fiber exhibited the highest density value.

### 3.3. Absorption Ratio

**Figure 7:**
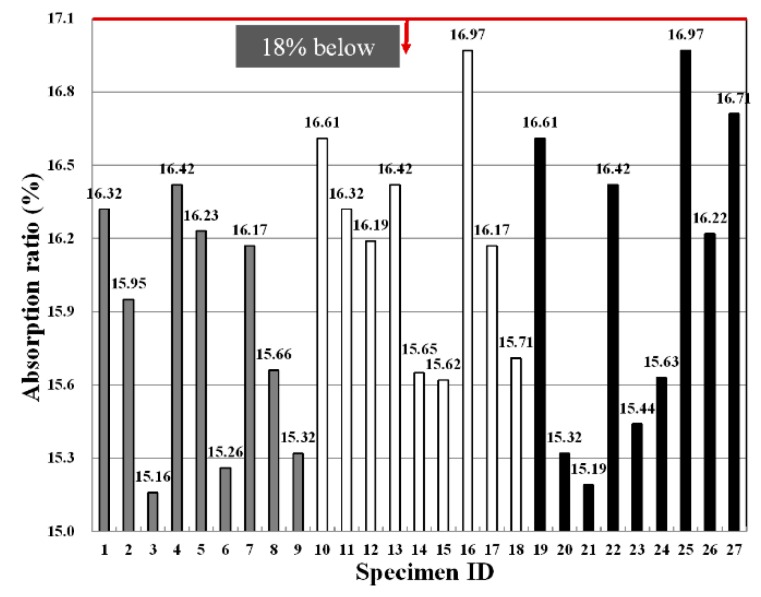
Variation of the absorption ratio for each specimen.

Figure 7 shows the absorption ratios depending on the fiber type, fiber mixing ratio and binder replacement ratio, and they were found to comply with standards in all experimental groups. In particular, the high fiber mixing ratio represents the lowest absorption ratio value.

This suggests that as the external extrusion panel is exposed to ambient air, a large amount of moisture is absorbed during rainy seasons, which in turn leads to an increase in load, cracking and discoloration and, thus, is known to be disadvantageous in terms of stability and aesthetics. Accordingly, a low absorption ratio is required for the external panels exposed to the outside air.

Table 10 shows F-test results for each factor obtained from the analysis of variance of absorption ratios. The results of the analysis of variance revealed that F-values of experimental factors like A (binder replacement rate), B (fiber type), C (fiber mixing ratio) were 3.537, 3.023 and 24.275, respectively. Since these experimental values were two in degrees of freedom and 16 in error and, thus, critical values F_0.01_, F_0.05_ and F_0.10_ were 6.23, 3.63 and 2.67 at confidence limits of 99% (significance level α = 0.01), 95% (significance level α = 0.05) and 90% (significance level α = 0.10), respectively, among the three experimental factors, A (binder replacement rate) and B (fiber type) were not significant even at 90% (significance level α = 0.10), whereas C (fiber mixing ratio) satisfied the confidence limit 99% (significance level α = 0.01) and thus was found to have a huge influence on the changes in absorption ratios.

**Figure 8:**
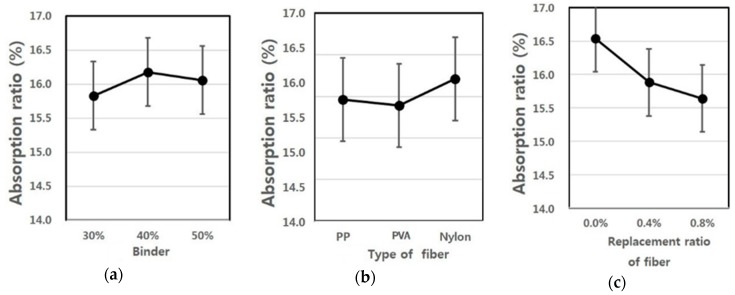
Assumed absorption ratio of extrusion panel by variance analysis: (**a**) replacement ratio of binder; (**b**) type of fiber; (**c**) replacement ratio of fiber.

A look at the changes in the absorption ratio due to the interaction revealed that as the degree of freedom is four and error is 16 and, thus, critical values F_0.01_, F_0.05_ and F_0.10_ were 4.77, 3.01 and 2.33 at confidence limits of 99% (significance level α = 0.01), 95% (significance level α = 0.05) and 90% (significance level α = 0.10), respectively, there was no interaction of A (binder replacement rate), B (fiber type) and C (fiber mixing ratio). However, the interaction between A (binder replacement rate) and B (fiber type) was found to affect the changes in abruption ratio, and its effects reached 95% (significance level α = 0.05). Figure 8 shows the absorption ratio values estimated using the test results, and the average value of the absorption ratio ranged from 15 to 16.

As shown in Figure 8, an increase in the binder mixing ratio did not significantly affect the changes in the absorption ratio, but the specimen using nylon fiber led to an increase in the abruption ratio with a slight difference compared to the specimen using PP and PVA fibers. Due to an increase in the fiber mixing ratio, the absorption ratio showed a great change, and as the fiber mixing ratio increased in the inside of the panel, the absorption ratio showed a tendency to decrease significantly.

### 3.4. Compressive Strength

**Figure 9:**
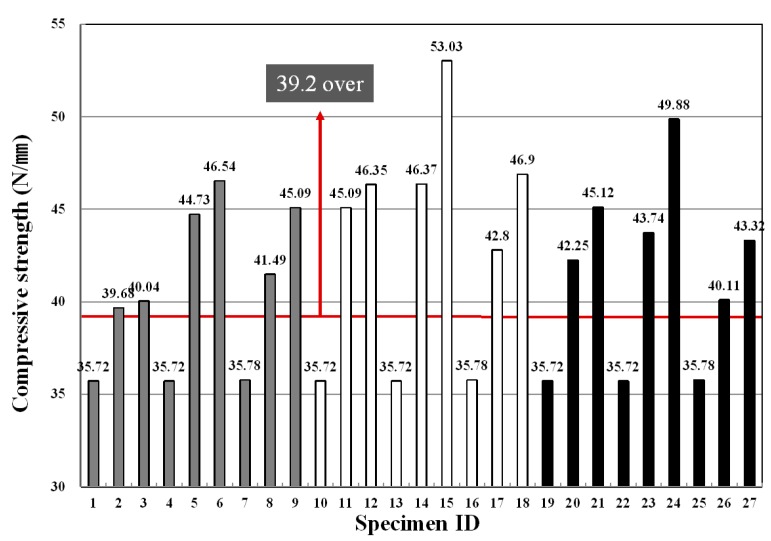
Variation of compressive strength for each specimen.

As shown in Figure 9, PP and PVA fibers showed similar compressive strengths, and nylon fiber exhibited a relatively low compressive strength value. However, the fiber type did not significantly affect the strength development. With respect to the fiber mixing ratio, the mixing ratio of 0.0% showed the lowest strength value, and as the mixing ratio increased from 0.4% to 0.8%, the strength increased, as well. This tendency is consistent with experimental results within the general mixing range of the conventional fiber reinforced concrete, and a similar tendency is also found in the extrusion panel [27,28,29].

Looking at the strength characteristics according to the binder replacement rate, the highest strength property was shown at the mixing ratio of 40%, whereas the strength development was more effective at the mixing ratio of 50% than at 30%, but was found to be lower than at the mixing ratio of 40%. In general, the ground granulated blast furnace slag is known to react to a stimulant, such as an alkali, without contributing to the initial hydration as a latent hydraulic material. Therefore, it has been reported that if steam cured rather than cured at room temperature, a large amount of calcium hydroxide is produced due to hydration promotion of cement inside the matrix, and the produced calcium hydroxide helps to activate the ground granulated blast furnace slag, thereby contributing to increasing the strength. Similar results to those of this experiment have been displayed [30]. For the CSA, ettringite is actively generated due to an increase in SO_3_-ion content and the rapid hydration of C_3_A (Tri-calcium aluminate) at an early age and, thus, contributes to increasing the strength [31].

Table 11 shows F-test results for each experimental factor obtained from the analysis of variance of compressive strength measurement values. The results of the analysis of variance revealed that F-values of experimental factors like A (binder replacement rate), B (fiber type) and C (fiber mixing ratio) were 8.911, 13.465 and 149.781, respectively. Additionally, since the experimental values were two in degrees of freedom and 12 in error and, thus, critical values F_0.01_, F_0.05_ and F_0.10_ were 6.93, 3.89 and 2.51 at confidence limits of 99% (significance level α = 0.01), 95% (significance level α = 0.05) and 90% (significance level α = 0.10), respectively, A (binder replacement rate), B (fiber type) and C (fiber mixing ratio) were all found to have met the 99% (significance level α = 0.01) level and, thus, turned out to be factors that exert a huge influence.

Looking at the changes in moisture content ratios due to the interaction, as the degree of freedom is four and error is 12 and, thus, critical values F_0.01_, F_0.05_ and F_0.10_ were 5.41, 3.26 and 2.48 at confidence limits of 99% (significance level α = 0.01), 95% (significance level α = 0.05) and 90% (significance level α = 0.10), respectively, among the three experimental factors, the interaction between A (binder replacement rate) and C (fiber mixing ratio) was significant at 90% (significance level α = 0.10); the influence of the interaction between B (fiber type) and C (fiber mixing ratio) on the compressive strengths was found to meet 95% (significance level α = 0.05); and there was no interaction between A (binder replacement rate) and B (fiber type).

**Figure 10:**
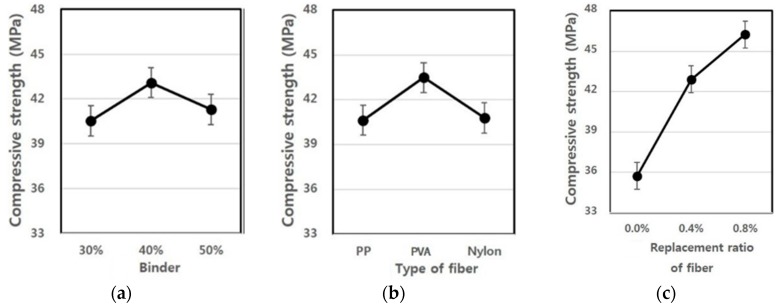
Assumed compressive strength of the extrusion panel by variance analysis: (**a**) replacement ratio of binder; (**b**) type of fiber; (**c**) replacement ratio of fiber.

Figure 10 shows changes in compressive strengths estimated using the test results, and the average value of the compressive strengths ranged from 35 to 46. As shown in the table, the binder mixing ratio of 40% showed the largest compressive strength value, and the specimen using PVA fiber exhibited a larger compressive strength development rate than the specimen using PP and nylon fibers. With an increase in the fiber mixing ratio, a change in the compressive strength development rate became great, and as the fiber mixing ratio inside the panel increased, the compressive strength showed a tendency to increase significantly.

### 3.5. Flexural Strength

**Figure 11:**
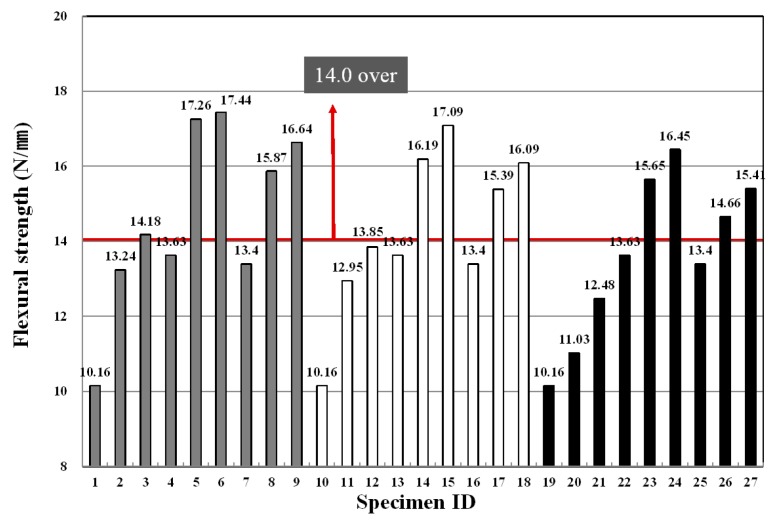
Variation of flexural strength for each specimen.

Similar to the characteristics of compressive strength, flexural strength demonstrated tendencies in which, as the binder replacement ratio and fiber mixing ratio increased, the flexural strength increased. In particular, the fiber mixing ratio was found to have a significant effect on the strength development. As shown in Figure 11, when the fiber mixing ratio was 0.0%, the flexural strength was 10.16 N/mm^2^, which did not meet the extrusion panel standards of more than 14.0 N/mm^2^, and as the mixing ratio increased from 0.4% to 0.8%, the flexural strength development rate increased significantly.

In addition, the flexural strength showed a similar tendency to the compressive strength according to the binder replacement rate. When the replacement rate was 40%, the flexural strength ranged from 14.18 to 17.44, exceeding the extrusion panel standards of more than 14.0 N/mm^2^. This result depends on the replacement rate of LA used for the purpose of excluding the autoclave curing, and suggests that a low replacement rate results from the lack of hydrate and hydration heat for the strength development inside the matrix.

Table 12 shows F-test results for each factor obtained from the analysis of variance of flexural strength results. The results of the analysis of variance found that F-values of experimental factors like A (binder replacement rate), B (fiber type) and C (fiber mixing ratio) were 22.113, 320.16 and 226.15, respectively.

Additionally, since the experimental values were two in degrees of freedom and 16 in error and, thus, critical values F_0.01_, F_0.05_ and F_0.10_ were 6.23, 3.63 and 2.67 at confidence limits of 99% (significance level α = 0.01), 95% (significance level α = 0.05) and 90% (significance level α = 0.10), respectively, A (binder replacement rate), B (fiber type) and C (fiber mixing ratio) all met the 99% (significance level α = 0.01) level and turned out to be factors that exert a huge influence.

**Figure 12:**
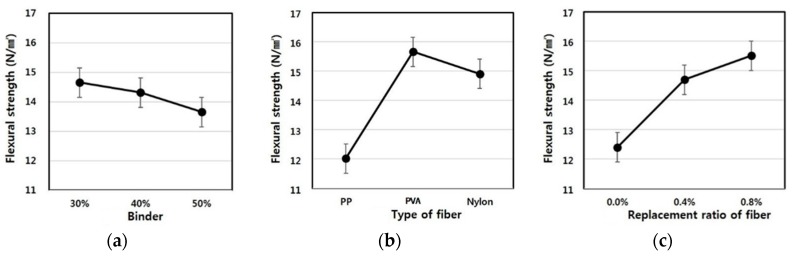
Assumed flexural strength of the extrusion panel by variance analysis: (**a**) replacement ratio of binder; (**b**) type of fiber; (**c**) replacement ratio of fiber.

Looking at the changes in densities due to the interaction revealed that as the degree of freedom is four and error is 16 and, thus, critical values F_0.01_, F_0.05_ and F_0.10_ were 4.77, 3.01 and 2.33 at confidence limits of 99% (significance level α = 0.01), 95% (significance level α = 0.05) and 90% (significance level α = 0.10), respectively, there was no interaction of A (binder replacement rate), B (fiber type) and C (fiber mixing ratio); however, the interaction between A (binder replacement rate) and C (fiber mixing ratio) had an influence on the change in absorption ratio, and these effects were found to meet 99% (significance level α = 0.01). Figure 12 shows the change in flexural strength estimated using the test results, and the average value of the flexural strength ranged from 12 to 16.

As demonstrated in the table, as the binder replacement rate increased, the flexural strength decreased. The results of the flexural strength development according to the fiber type showed that the specimen using PE fibers exhibited a greater flexural strength development ratio than the specimen using PP and nylon fibers, and the fiber mixing ratio also yielded the same results.

## 4. Summary and Conclusions

In this study, an experiment was conducted with respect to the binder replacement rate, fiber type and fiber mixing ratio in order to reduce the amount of curing in the production phase of extrusion panels, which are secondary products of concrete, and the following conclusions were obtained from the experimental results.

- According to the analysis of variance of the moisture content ratios and densities according to the binder replacement rate, fiber type and fiber mixing ratio, the fiber type and fiber mixing ratio turned out to be the most effective factors, and the influence of the binder mixing ratio was found to have decreased slightly.
- The analysis of variance of the absorption ratio found that the fiber mixing ratio was an effective factor that has a huge influence on the absorption ratio changes, while the influence of the binder mixing rate and fiber type was small.
- The analysis results of the flexural and compressive strengths showed that the binder replacement rate, fiber type and fiber mixing ratio were all effective factors that exert a great influence on the strength development, and the interaction on the binder replacement rate and fiber type was lacking.

The above experimental and empirical research results suggest that the target properties of the cement extrusion panels are satisfied by the binder replacement rate of 40%, the use of low-cost PR and nylon fibers rather than PP and the fiber mixing ratio of at least 0.8% or more, and further research needs to be conducted regarding the long-term durability, resistance to outdoor exposure and freeze-thaw resistance. Finally, the proposed modified curing method can be applied to manufacture the extrusion panel in order to reduce CO_2_ emission in the construction material industry. Therefore, it will be helpful to reduce global warming.

## Figures and Tables

**Table 1:** Experimental plan.

Factors	Levels			Test Items
Replacement ratio of LA (%)	30%	40%	50%	- Compressive strength - Flexural strength - Absorption ratio - Density - Moisture content ratio
Type of fiber	PP	PVA	Nylon	
Replacement ratio of fiber (%)	0.0%	0.4%	0.8%	

**Table 2:** Characteristics of fibers.

Name	Specific Gravity (kg/cm^3^)	Length (mm)	Diameter (mm)	Aspect Ratio	Tensile Strength (MPa)	Modulus of Elasticity (GPa)
PP	0.96	15	0.012	1250	5600	5.0
PVA	1.3	15	0.015	1000	1269	2.7
Nylon	1.1	13	0.023	565	919	5.3

**Table 3:** Mix conditions for the experimental test.

ID	Type of Fiber	Replacement Ratio of LA (%)	Replacement Ratio of Fiber (%)
1	PP	30%	0.0%
2			0.4%
3			0.8%
4		40%	0.0%
5			0.4%
6			0.8%
7		50%	0.0%
8			0.4%
9			0.8%
10	PVA	30%	0.0%
11			0.4%
12			0.8%
13		40%	0.0%
14			0.4%
15			0.8%
16		50%	0.0%
17			0.4%
18			0.8%
19	Nylon	30%	0.0%
20			0.4%
21			0.8%
22		40%	0.0%
23			0.4%
24			0.8%
25		50%	0.0%
26			0.4%
27			0.8%

**Table 4:** Physical and chemical properties of OPC.

Setting Time (min)		Density (g/cm^3^)	Blain (cm^2^/g)	LOI(Loss on Ignition)	Chemical Component (%)							LSF (Lime Saturation Factor)
Start	Finish				SiO_2_	Al_2_O_3_	Fe_2_O_3_	CaO	MgO	SO_3_	K_2_O	
290	380	3.15	3240	0.35	LSF	5.02	3.66	64.18	2.01	1.83	0.92	90.4

**Table 5:** Physical properties of fine aggregate.

F.M. (Fineness Modulus)	Density (g/cm^3^) Dry	Absorption Ratio (%)	Percentage of Solid Volume (%)
1.82	2.51	1.32	44.6

**Table 6:** Chemical properties of materials.

Name	Blain (cm^2^/g)	Chemical Component (%)								Ig-Loss (Ignition Loss)
		SiO_2_	Al_2_O_3_	Fe_2_O_3_	CaO	MgO	Na_2_O	SO_3_	K_2_O	
Blast furnace slag	7400	33.4	15.8	0.6	41.8	5.3	0.3	1.5	0.3	-
CSA	4500	4.14	36.5	0.4	38.17	3.00	0.11	6.14	11.02	0.52
CK	4200	52.1	41.0	4.32	0.07	0.19	0.31	-	0.58	-
CAMC	4200	7.0	21.0	0.5	50.0	4.43	-	3.03	-	-

**Table 7:** Test results.

ID	Type of Fiber	Replacement Ratio of LA (%)	Replacement Ratio of Fiber (%)	Moisture Content Ratio (%)	Absorption Ratio (%)	Density (g/cm^3^)	Compressive Strength (N/mm^2^)	Flexural Strength (N/mm^2^)
1	PP	30	0.0	1.05	16.32	1.78	35.72	10.16
2			0.4	1.89	15.95	1.76	39.68	13.24
3			0.8	1.49	15.16	1.74	40.04	14.18
4		40	0.0	1.26	16.42	1.73	35.72	13.63
5			0.4	0.54	16.23	1.71	44.73	17.26
6			0.8	0.28	15.26	1.67	46.54	17.44
7		50	0.0	3.6	16.17	1.62	35.78	13.4
8			0.4	2.42	15.66	1.59	41.49	15.87
9			0.8	1.33	15.32	1.58	45.09	16.64
10	PVA	30	0.0	1.05	16.61	1.78	35.72	10.16
11			0.4	2.31	16.32	1.78	45.09	12.95
12			0.8	1.31	16.19	1.77	46.35	13.85
13		40	0.0	1.26	16.42	1.73	35.72	13.63
14			0.4	1.23	15.65	1.67	46.37	16.19
15			0.8	1.18	15.62	1.66	53.03	17.09
16		50	0.0	3.6	16.97	1.64	35.78	13.4
17			0.4	3.23	16.17	1.58	42.8	15.39
18			0.8	1.18	15.71	1.38	46.9	16.09
19	Nylon	30	0.0	1.05	16.61	1.78	35.72	10.16
20			0.4	2.85	15.32	1.77	42.25	11.03
21			0.8	2.31	15.19	1.73	45.12	12.48
22		40	0.0	1.26	16.42	1.67	35.72	13.63
23			0.4	2.21	15.44	1.65	43.74	15.65
24			0.8	1.84	15.63	1.63	49.88	16.45
25		50	0.0	3.6	16.97	1.62	35.78	13.4
26			0.4	2.28	16.22	1.58	40.11	14.66
27			0.8	1.02	16.71	1.35	43.32	15.41

**Table 8:** Variance analysis for the moisture content ratio of the extrusion panel.

ID	Factors	S	Ø	V	F_0_	Evaluation
A	Replacement ratio of binder	1.159	2	0.579	4.158	**
B	Type of fiber	7.104	2	3.552	25.490	***
C	Replacement ratio of fiber	3.123	2	1.561	11.208	***
A*B		1.410	4	0.353	2.530	-
A*C		8.432	4	2.108	15.130	***
Error		1.672	12	0.139		
Total		22.899	26			

S: Sum of squares; Ø: Degree of Freedom; V: Mean of the sum of squares; F_0_: F-statistics value; *** Accepted at the 0.01 significance level; ** accepted at the 0.05 significance level; * accepted at the 0.10 significance level; -, not accepted.

**Table 9:** Variance analysis for the dry density of the extrusion panel.

ID	Factors	S	Ø	V	F_0_	Evaluation
A	Replacement ratio of binder	0.009	2	0.004	2.462	
B	Type of fiber	0.214	2	0.107	59.331	***
C	Replacement ratio of fiber	0.041	2	0.021	11.375	***
B*C		1.410	4	0.006	3.462	**
Error		1.672	16	0.002		
Total		0.3183	26			

*** Accepted at the 0.01 significance level; ** accepted at the 0.05 significance level; * accepted at the 0.10 significance level.

**Table 10:** Variance analysis for absorption ratio of the extrusion panel.

ID	Factors	S	Ø	V	F_0_	Evaluation
A	Replacement ratio of binder	0.572	2	0.286	3.537	-
B	Type of fiber	0.489	2	0.245	3.023	-
C	Replacement ratio of fiber	3.928	2	1.964	24.275	***
B*C		1.514	4	0.378	4.677	**
Error		1.294	16	0.081		
Total		7.797	26			

*** Accepted at the 0.01 significance level; ** accepted at the 0.05 significance level; * accepted at the 0.10 significance level; -, not accepted.

**Table 11:** Variance analysis for the compressive strength of the extrusion panel.

ID	Factors	S	Ø	V	F_0_	Evaluation
A	Replacement ratio of binder	30.903	2	15.452	8.911	***
B	Type of fiber	46.696	2	23.348	13.465	***
C	Replacement ratio of fiber	519.438	2	259.719	149.78	***
A*B		19.935	4	4.984	2.874	-
A*C		32.559	4	8.139	4.694	**
Error		20.808	12	1.734		
Total		670.339	26			

*** Accepted at the 0.01 significance level; ** accepted at the 0.05 significance level; * accepted the 0.10 significance level; -, not accepted.

**Table 12:** Variance analysis for the flexural strength of the extrusion panel.

ID	Factors	S	Ø	V	F_0_	Evaluation
A	Replacement ratio of binder	4.596	2	2.298	22.113	***
B	Type of fiber	66.549	2	33.275	320.16	***
C	Replacement ratio of fiber	47.008	2	23.504	226.15	***
A*C		2.401	4	0.600	5.776	***
Error		1.663	16	0.104		
Total		7.797	26			

*** Accepted at the 0.01 significance level; ** accepted at the 0.05 significance level; * accepted at the 0.10 significance level.

## Abbreviations

The following abbreviations are used in this manuscript:

LOI	Loss On Ignition
OPC	Ordinary Portland cement
LSF	Lime saturation factor
F.M.	Fineness Modulus
CK	Calcined Kaolin
Ig-Loss	Ignition Loss
LA	Low energy curing admixture
PPM	Parts per million
PVA	Polyvinyl acetate
RH	Relative humidity
CSA	Calcium sulfate aluminate
PP	Polypropylene
CAC	Calcium aluminate compound
CSA	Calcium sulfate aluminate
C_3_A	Tri-calcium aluminate
CAMC	Calcium aluminate modified composites
CP	Centipoise
S	Sum of squares
Ø	Degree of freedom
V	Mean of the sum of squares
F_0_	F-statistics value
